# Climate‐Driven Spatiotemporal Dynamics of Potential Distribution for *Nanorana quadranus* in China

**DOI:** 10.1002/ece3.74131

**Published:** 2026-08-17

**Authors:** Yuan Sun, Lulu Lyu, Jie Peng, Yan Huang

**Affiliations:** ^1^ Key Laboratory of Southwest China Wildlife Resources Conservation (Ministry of Education) China West Normal University Nanchong China; ^2^ Key Laboratory of Artificial Propagation and Utilization in Anurans of Nanchong City China West Normal University Nanchong China

**Keywords:** climate change, conservation planning, frog, habitat suitability, species distribution models (SDMs)

## Abstract

Global climate change is accelerating the loss of biodiversity worldwide. The Qinling and Daba Mountains represent National Key Ecological Function Zones in China where complex environmental gradients render resident fauna sensitive to climatic fluctuations. However, the specific spatiotemporal dynamics of endemic species in this region in response to these shifts remain largely unquantified. *Nanorana quadranus* , a Near Threatened amphibian endemic to this region, serves as an ideal ecological indicator species due to its reliance on cold‐water mountain streams and its sensitivity to thermal and hydrological fluctuations. In this study, the optimized Maxent was utilized to predict the current and future suitable habitat distribution of *N. quadranus* under the SSP2‐4.5 and SSP5‐8.5 scenarios. Additionally, the biomod2 ensemble framework was employed for independent spatial validation. By analyzing the relationship between geographic distribution and bioclimatic variables, it was revealed that the Minimum Temperature of the Coldest Month (Bio6) is the most important predictor (42.9% Percent Contribution, 75.9% Permutation Importance), suggesting a strong statistical association between Bio6 and the potential distribution of this species. Model results indicate that under current climate conditions, the total suitable habitat area reaches 39.95 × 10^4^ km^2^, primarily distributed in the Qinling‐Daba Mountains of central and southwestern China. Future projections indicate a continuous and severe contraction of suitable habitats for *N. quadranus* , with habitat loss substantially exceeding habitat gain. Furthermore, the centroid of suitable habitat is projected to shift northwestward by 417.3 km by the end of this century under the SSP5‐8.5 scenario. The results of this study not only quantified the current and future potential suitable habitat distribution of *N. quadranus* , but also provided scientific measures for the conservation of this Near Threatened species.

## Graphical abstract

This study integrates an ensemble modeling framework and an optimized Maxent model to assess the current and future habitat suitability of *Nanorana quadranus* under climate change scenarios. Results indicate that temperature‐related variables, particularly the minimum temperature of the coldest month (Bio6), are the most important predictor of species distribution, while future projections reveal substantial habitat contraction and a pronounced northwestward shift. This study provides a scientific basis for the conservation planning of this endemic species and offers a reference for the protection of other amphibians inhabiting cold‐water stream habitats in the Qinling‐Daba Mountains.

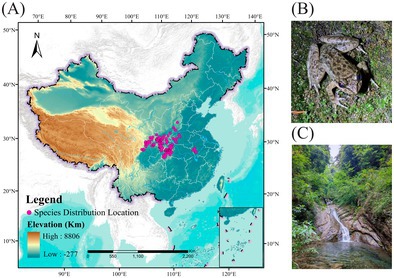

## 1 Introduction

Driven by global climate change, the rapid alteration of environmental conditions is triggering shifts in suitable habitats of species, thereby accelerating global biodiversity loss (Pecl et al. 2017). Mountain ecosystems, characterized by complex environmental gradients, are particularly vulnerable to these climatic fluctuations (Zeng et al. 2025). The Qinling and Daba Mountains of China represent not only one of the National Key Ecological Function Zones in China but also a vital transitional zone between the northern and southern China. This unique geographical environment has created diverse climatic conditions and abundant local habitats, but the complex topographical features have also become natural barriers for species to spread (Ghalambor et al. 2006; Zheng Zhi et al. 2014). Many species in mountainous ecosystems are likely more vulnerable under climate change (Loarie et al. 2009). Predicting climate‐driven shifts in suitable habitats of endemic mountain species is crucial for developing effective local conservation measures.

Because of their permeable skin and metamorphic life cycle, amphibians are sensitive to temperature and hydrological changes, and thus are considered key indicators of ecosystem health (Longhini et al. 2021; Womack et al. 2022). *Nanorana quadranus* (Anura: *Dicroglossidae*) is a mountain frog endemic to China, distributed in cold mountain streams and adjacent pools of the Qinling‐Daba Mountains at elevations of 335 to 1830 m. Its survival is constrained by specific thermal and hydrological thresholds, with its distribution confined to the cold‐water streams of this region (Collins et al. 2009; Fei et al. 2012). The reproduction and development of *N. quadranus* are strongly contingent upon specific stream microhabitat conditions, including water temperature and flow velocity (Lyu et al. 2025). Global warming is altering the hydrological conditions and temperature regimes of mountain streams (Alves‐Ferreira et al. 2022), thereby modifying the habitats upon which this amphibian species depends. *N. quadranus* is currently listed as Near Threatened (NT) on the China Red List and is confronting progressively more severe threats to its survival (Jiang et al. 2016). Existing research on *N. quadranus* has largely focused on morphology, reproductive biology, and local population surveys (Fu et al. 2006; Lyu et al. 2025). While these traditional field surveys have provided indispensable empirical data on local ecologies, there remains a critical need to build upon these baseline records to predict the long‐term spatial dynamics of this species at a macro scale. Therefore, it is essential to integrate occurrence data from field observations with predictive modeling to quantify its spatial responses to future climate scenarios.

Species Distribution Models (SDMs) employ computational algorithms to quantify statistical associations between species occurrence records and environmental variables, thereby estimating the geographic distribution of species under prevailing environmental conditions. They are important tools for predicting geographic ranges and identifying environmental predictors (Elith and Leathwick 2009). As ectotherms, amphibians are physiologically regulated by ambient temperature and humidity. This physiological dependence renders them particularly sensitive to macroclimatic shifts, motivating the widespread application of SDMs in amphibian research. In amphibian research, SDMs are primarily used to explore the distribution of species and evaluate the impacts of climate change. For instance, a global‐scale study utilizing SDMs for amphibians predicted that the combined impacts of climate change and other stressors will disproportionately affect regions harboring the highest amphibian species richness (Hof et al. 2011). Among SDMs, the Maximum Entropy model (Maxent) is widely used in habitat suitability studies owing to its robust performance with presence‐only data (Phillips et al. 2006). However, like all SDMs, its predictive accuracy is contingent upon the quality of occurrence records and environmental data. Following rigorous scientific data screening, Maxent has been widely applied in amphibian research. For instance, it has been used to predict the potential distribution ranges of *Rana chensinensis* and *Quasipaa spinosa* , thereby informing the development of targeted conservation measures (Hou et al. 2023; Fu et al. 2025).

In this study, we first optimized the parameter settings of the Maxent model using the ENMeval package to enhance its predictive performance. Subsequently, by integrating environmental variables with species occurrence data, we applied the optimized Maxent model to identify key environmental predictors and to simulate the potential suitable habitats of *N. quadranus* . Concurrently, we constructed an ensemble model using the biomod2 platform to evaluate spatial consistency. This ensemble model included generalized linear models (GLM), generalized boosted models (GBM), generalized additive models (GAM), random forest (RF), and optimized Maxent. Specifically, this research aims to address three core questions, (1) Identify the dominant environmental factors that currently restrict the distribution of *N. quadranus* ; (2) Predict the spatiotemporal dynamics of suitable habitats under future climate change scenarios; (3) Evaluate whether the distribution centroid shift predicted is beyond the natural dispersal capacity of the species. The results of this study not only fill a research gap regarding how the potential distribution of this species responds to climate change, but also provide a preliminary reference for the conservation of this species under future climate change.

## 2 Materials and Methods

### 2.1 Species Occurrence Data Collection and Processing

This study follows the standard process of species distribution modeling to increase the prediction accuracy and model generality (Araújo et al. 2019). A total of 1406 occurrence records for *N. quadranus* were initially compiled from three sources: (1) field surveys conducted by our research team between 2018 and 2025 across the Qinling‐Daba Mountains using visual encounter surveys along stream transects yielded a total of 368 presence records (Table S5); (2) the Global Biodiversity Information Facility (GBIF, https://doi.org/10.15468/dl.788ct4; accessed on October 10, 2025), providing 1013 records of *N. quadranus* from China; and (3) related literature searched from the China National Knowledge Infrastructure (CNKI), yielding 25 occurrence records.

**FIGURE 1:**
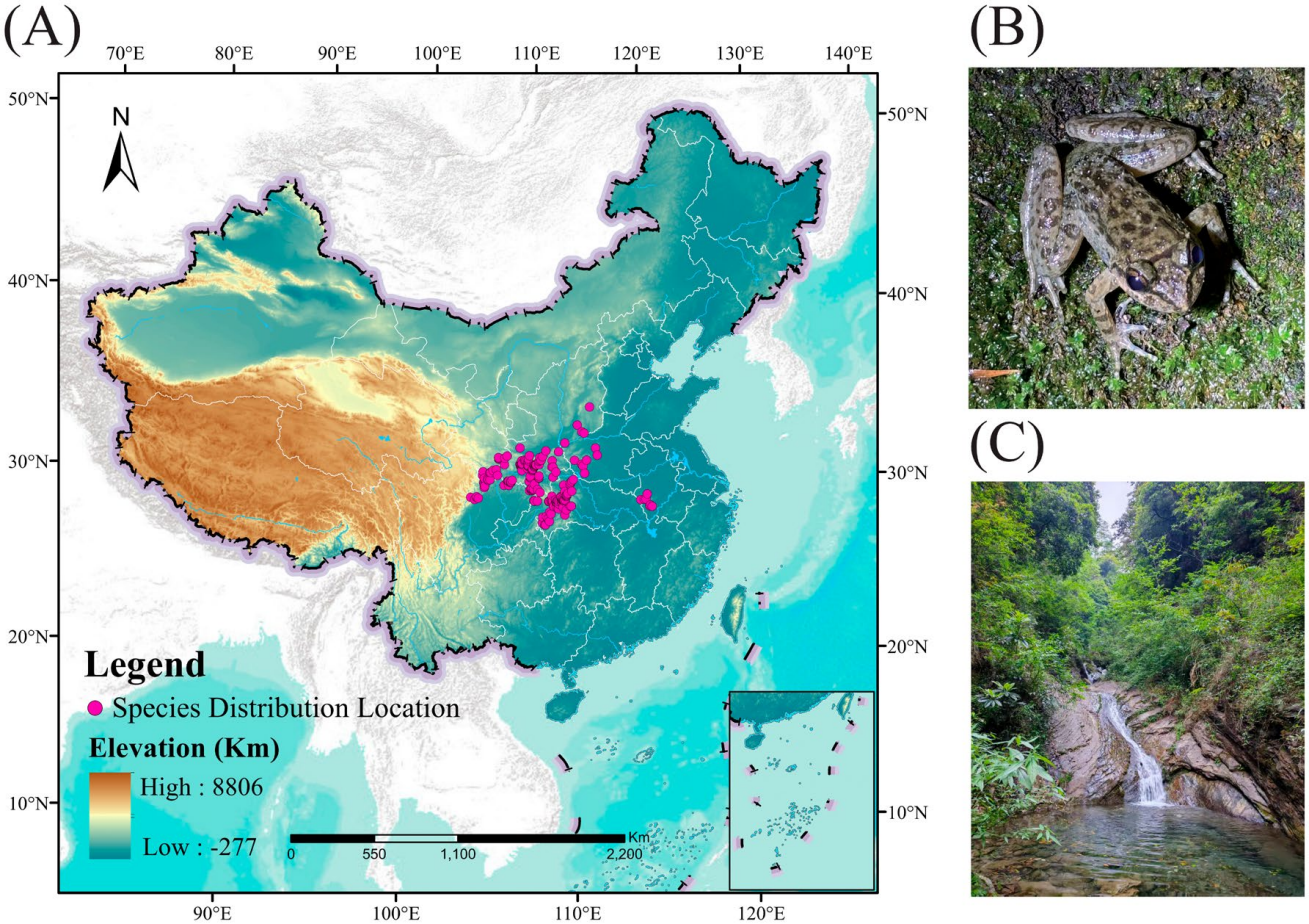
Species Distribution Location of *Nanorana quadranus* in China. (A) Overview of the occurrence points at the national level; (B) Adult individual of *N. quadranus* (Photographed in Micangshan Nature Reserve, Sichuan Province, by Lulu Lyu); (C) Typical natural habitat of *N. quadranus* (Photographed in Mianyang City, Sichuan Province, by Yuan Sun).

Occurrence data collected from multiple sources may be subject to quality issues, such as spatial uncertainty, taxonomic conflicts, and misidentification (Tessarolo et al. 2021). In order to weaken the effects caused by sample bias and spatial autocorrelation (Beck et al. 2014; Fourcade et al. 2014; Phillips et al. 2009), we used ENMTools (Warren et al. 2010) to process occurrence data. To eliminate spatial redundancy and sampling bias, occurrence data were spatially rarefied using ENMTools, ensuring that only one presence record was retained within each 2.5 arc‐minute environmental raster cell. This 2.5 arc‐minute resolution represents a standard intermediate choice that balances data availability, computational efficiency, and ecological relevance for regional‐scale species distribution modeling (Aiello‐Lammens et al. 2015; Boria et al. 2014). We confined the records to the geographical boundaries of China, removed duplicates and incomplete entries, and excluded spatially autocorrelated points, thereby retaining 193 high‐quality occurrence records for subsequent modeling analyses (Figure 1).

### 2.2 Environmental Variables

A total of 27 environmental variables were selected based on the ecological characteristics of *N. quadranus* from four categories: climate, topography, soil, and human activities. Among them, there are 19 bioclimatic variables (Bio1–Bio19) based on the WorldClim database (http://www.worldclim.org/; accessed on 13 October 2025), with a spatial resolution of 2.5 arc minutes. Two SSPs (SSP2‐4.5 and SSP5‐8.5) were selected from the BCC‐CSM2‐MR model under the CMIP6 framework. The BCC‐CSM2‐MR model developed by the Beijing Climate Center (BCC) of China was selected because it has demonstrated reliable performance in simulating temperature and precipitation over East Asia, particularly over China (Li et al. 2023). The SSP2‐4.5 scenario assumes moderate greenhouse gas emissions, reflecting a future where social, economic, and technological trends do not change significantly from historical patterns; the SSP5‐8.5 scenario assumes the highest greenhouse gas emissions, reflecting a future of high fossil‐fuel use for economic growth with little environmental consideration (Riahi et al. 2017). Based on these two scenarios, we predicted four future stages (the 2030s: 2021–2040; the 2050s: 2041–2060; the 2070s: 2061–2080; the 2090s: 2081–2100) (Thomas et al. 2004).

Topographic predictors (elevation, slope, and aspect) were sourced from WorldClim, and soil‐related predictors (topsoil pH, Soil Type, available water capacity, and percentage of organic carbon) were obtained from the Harmonized World Soil Database (HWSD; http://www.fao.org/), accessed on 13 October 2025 (Austin and Van Niel 2011; Körner 2007). The level of human disturbance was instead represented by the Global Terrestrial Human Footprint (HF) index (Venter et al. 2016). Since they are considered relatively stable over longer temporal scales compared with climate predictors (Elith et al. 2011; Hannah et al. 2007), we treated all topographic and soil predictors as fixed covariates for all the future time steps.

**FIGURE 2:**
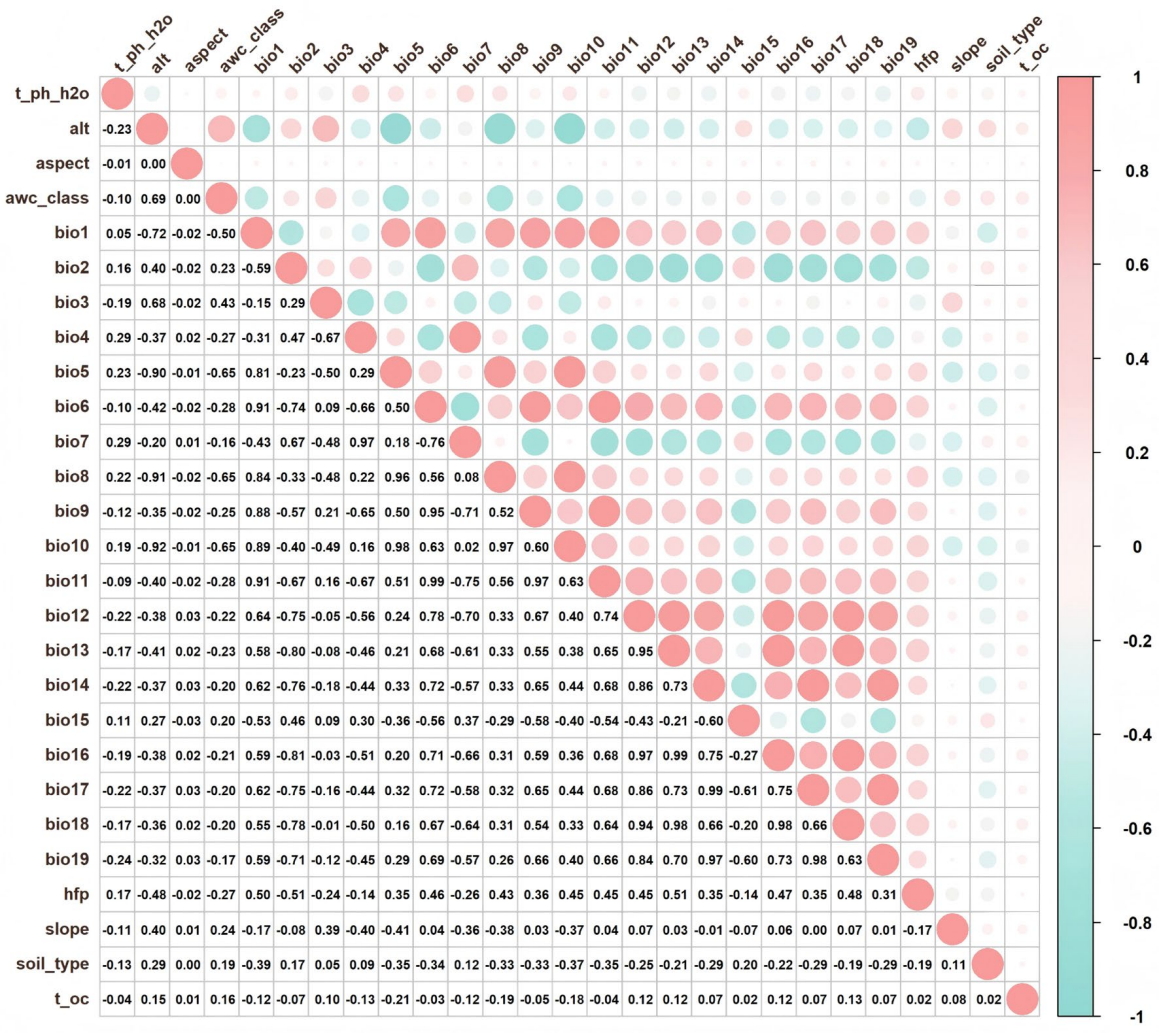
Pearson correlation heatmap of the initial 27 environmental variables. The color scale on the right indicates the values of the correlation coefficient, ranging from blue‐green (negative correlation, −1) to carmine red (positive correlation, 1). The numbers in the grid represent specific Pearson r values. This matrix is used to identify and remove highly correlated predictors to reduce multicollinearity in the modeling process.

To minimize multicollinearity and model complexity (Chollet Ramampiandra et al. 2023; Júnior and Nóbrega 2018), we conducted a two‐stage screening procedure. First, we computed Pearson's correlation coefficient based on ENMTools and drew a correlation heatmap in *R* using the corrplot package (Figure 2). Variable pairs with high collinearity ($\left|r\right|\geq 0.8$) were removed, and we only retained one variable of greater ecological significance (Díaz‐Vallejo et al. 2024; Schnase and Carroll 2022). Second, we ran a trial Maxent model to evaluate the importance of variables according to Percent Contribution (PC), Permutation Importance (PI), and the Jackknife test (Elith et al. 2011). Variables with negligible contributions (< 1%) were excluded to prevent model overfitting and eliminate uninformative predictors. Nine predictor variables finally remained: Bio2 (Mean diurnal range), Bio3 (Isothermality), Bio4 (Temperature seasonality), Bio6 (Minimum temperature of coldest month), Bio10 (Mean temperature of warmest quarter), Bio12 (Annual precipitation), Bio15 (Precipitation seasonality), Slope, and Soil Type.

### 2.3 Species Distribution Modeling and Model Optimization

In this study, we first used the ENMeval package in *R* to optimize the parameters of Maxent, thereby enhancing its generalization ability and reducing overfitting (Muscarella et al. 2014). The Maxent model was optimized by tuning two key parameters, feature class (FC) and regularization multiplier (RM), to achieve an optimal balance between model complexity and predictive performance (Warren et al. 2021). We tested 48 parameter combinations (Table S1), consisting of six FC combinations (L, LQ, H, LQH, LQHP, LQHPT) and eight RM values (RM = 0.5 to 4.0, step size 0.5) (Phillips et al. 2006). Finally, we selected the optimal models based on the lowest corrected Akaike Information Criterion (AICc), which offers a more balanced trade‐off between model complexity and goodness of fit (Burnham and Anderson 2002; Warren and Seifert 2011).

Meanwhile, to independently validate the predictions generated by the optimized Maxent model, we employed the biomod2 package (v4.2) in *R* (v4.4.1) to train five distinct machine learning models, namely GLM, GAM, GBM, RF, and optimized Maxent. Identical occurrence records and environmental covariates were used across all five algorithms to ensure comparability. The predictive performance of the five models was evaluated using two standard metrics commonly used in species distribution modeling: the Area Under the Curve (AUC) and the True Skill Statistic (TSS). Furthermore, to explore the agreement of variable importance across different modeling algorithms, we extracted permutation‐based variable importance scores for each of the five algorithms (GLM, GAM, GBM, RF, and Maxent) from the biomod2 framework. The relative importance of the top five environmental predictors for each algorithm was subsequently visualized using a chord diagram. Finally, to enhance prediction reliability, only single models achieving a True Skill Statistic (TSS) threshold of 0.7 or higher were retained for ensemble model construction (Allouche et al. 2006). The spatial distribution patterns produced by the ensemble model were visually compared against those generated by the optimized Maxent model to evaluate their spatial consistency. Notably, this ensemble model was employed solely as an independent spatial reference for validating the optimized Maxent results; it was not utilized for subsequent predictive modeling or environmental variable identification.

For final prediction and environmental factor identification, we used the optimized Maxent model. The model was constructed using the subsampling method with 10 replicate runs; in each run, we randomly selected 75% of the occurrence records as the training dataset and the remaining 25% as the test dataset (Phillips et al. 2006; Shabani et al. 2018). This repeated subsampling procedure enhances the robustness of model generalizability assessment and yields more stable and reliable estimates of predictive performance (Merow et al. 2013). Model performance was primarily evaluated using AUC values. AUC values (≥ 0.9) indicate excellent predictive ability, while values between 0.7 and 0.9 suggest good performance (Geurts et al. 2023; Valavi et al. 2023). The relative contribution of each environmental predictor was quantified using three measures: PC, PI, and the Jackknife test (Wang et al. 2025; Zhang et al. 2024).

### 2.4 Spatiotemporal Dynamic Analysis

The habitat suitability map was converted into a binary map according to the Maximum Test Sensitivity Plus Specificity (MTSPS) threshold value of 0.1733 (Liu et al. 2013). Based on this threshold, habitats were classified into four categories: Unsuitable (< 0.1733), Low Suitability (0.1733–0.4), Moderate Suitability (0.4–0.6), and High Suitability (> 0.6). Areas were calculated based on the binary MTSPS (0.1733), while the four‐level classification was used for visualization.

Spatiotemporal changes in suitable habitats were analyzed in ArcGIS by overlaying future predictions onto the current distribution. Changes were classified into three categories: stable areas (persistently suitable), lost areas (currently suitable but projected to become unsuitable), and gained areas (currently unsuitable but projected to become suitable). SDMtoolbox v2.6 in ArcGIS was implemented to compute the geometric centroid of suitable habitat areas across multiple future time periods (Brown 2014). Finally, *R* was employed to quantify the magnitude and trajectory of centroid shifts, visualizing the spatiotemporal dynamics of *N. quadranus* .

## 3 Results

### 3.1 Model Accuracy, Dominant Factors and Environmental Factor Identification

**FIGURE 3:**
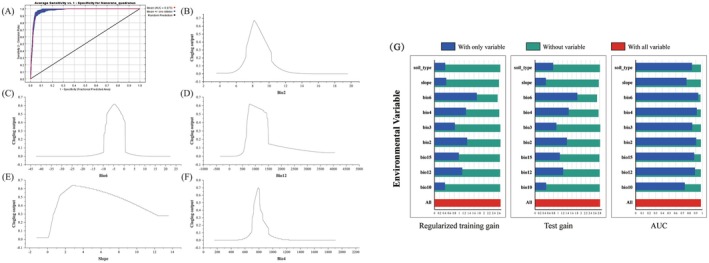
Maxent model performance, response curves of environmental predictors, and jackknife test results. (A) Model performance was evaluated using the Receiver Operating Characteristic (ROC) curve and the Area Under the Curve (AUC), with higher AUC values indicating stronger predictive performance; (B) Bio2, Mean Diurnal Range; (C) Bio6, Min Temperature of Coldest Month; (D) Bio12, Annual Precipitation; (E) Slope, Slope; (F) Bio4, Temperature Seasonality; (G) Jackknife test results for environmental variables.

The optimized Maxent algorithm was selected for subsequent optimization and environmental factor identification. The optimal settings obtained through ENMeval were RM = 1.0 and FC = LQHP (Table S1), yielding a Test AUC value of 0.977 (Figure 3A).

**FIGURE 4:**
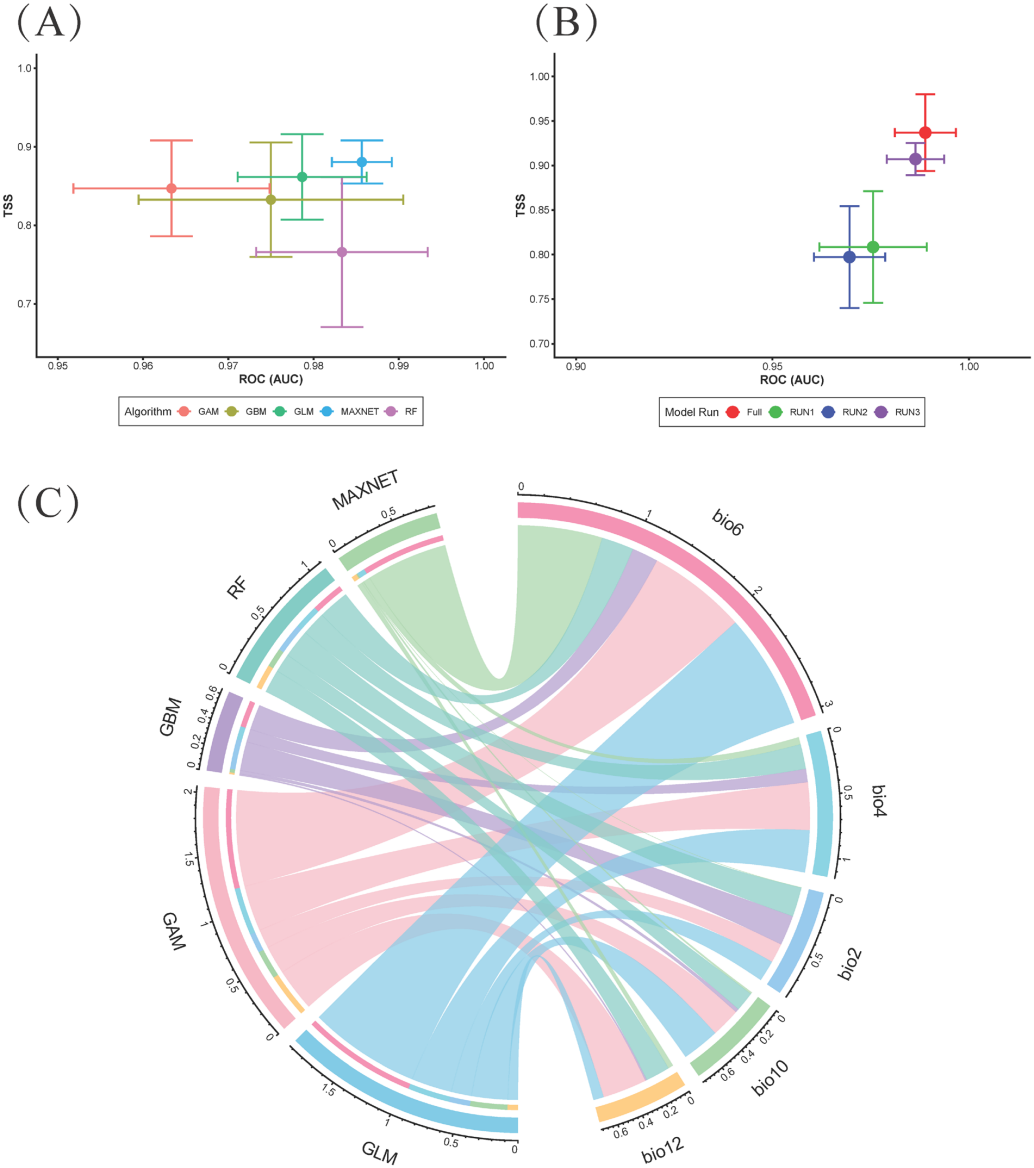
Performance assessment and relative variable contributions of the five modeling algorithms. (A) Predictive performance comparison of five algorithms (mean ± SD, AUC vs. TSS); (B) Model robustness evaluation across different data partitions (Full model vs. RUN1–3); (C) Relative importance of top five environmental variables across algorithms: Chord width indicates variable contribution.

The cross‐validation results of the multialgorithm framework in biomod2 showed that prediction performance was consistent across all five algorithms (GLM, GAM, GBM, RF, and optimized Maxent), with mean AUC values above 0.96 and mean TSS values above 0.75 (Table 1; Figure 4A). In terms of prediction stability across repeated validation runs, the optimized Maxent algorithm showed the highest stability compared with other models with a mean AUC of 0.986 ± 0.004 and a mean TSS of 0.881 ± 0.027. From the consistency check part, we found a good match between the full model and each of the three repeated validation runs (RUN1–RUN3) (Figure 4B).

**TABLE 1:** Predictive performance of five modeling algorithms for *N. quadranus* . Values are presented as mean ± standard deviation (SD) based on 3‐fold cross‐validation.

algo	AUCroc	TSS
GAM	0.963 ± 0.012	0.847 ± 0.061
GBM	0.975 ± 0.016	0.833 ± 0.073
GLM	0.979 ± 0.008	0.862 ± 0.054
MAXENT	0.986 ± 0.004	0.881 ± 0.027
RF	0.983 ± 0.010	0.766 ± 0.095

The multialgorithm chord diagram (Figure 4C) revealed consistent importance rankings of environmental predictors across different algorithms. In particular, Bio6 and Bio4 showed relatively higher importance under all five algorithms. Based on the optimized Maxent model, we found differences in both PC and PI values among environmental factors (Table 2). Temperature‐related variables were the major predictors, with Bio6 showed the highest PC (42.9%) and PI (75.9%), followed by Bio2 (PC = 18%; PI = 0.4%). Among nonclimatic predictors, slope had the highest PC of 10.1% and PI of 6.2% values. Bio4 and Bio3 had PC values of 10% and 8.8%, and PI values of 7.2% and 1%, respectively. The Bio10 had PC values of 4.3% and PI values of 3.7%. The remaining variables showed low importance, except for Bio15 (PC = 3.5%; PI = 2.7%) and Bio12 (PC = 1.4%; PI = 2.5%). Soil type showed relatively minor contributions (PC = 0.8%; PI = 0.3%). Jackknife analysis (Figure 3G) identified Bio6 as the most influential predictor of the potential distribution of species. It achieved the highest gain when only this variable was included and caused the greatest decline in model performance when excluded. Other temperature‐related factors, especially Bio4 and Bio2, also made relatively high contributions to the explanatory power of model, while topographic and soil variables played a secondary role.

**TABLE 2:** Percent contribution (PC) and permutation importance (PI) of the final environmental variables in the Maxent model. The table presents the nine optimal environmental variables retained for model construction and their contribution metrics from the preliminary runs, reflecting their relative importance in shaping the potential distribution of species.

Environmental variables	Description	Unit	PC (%)	PI (%)
Bio6	Min temperature of coldest month	°C	42.9	75.9
Bio2	Mean diurnal range	°C	18	0.4
Slope	Slope	°	10.1	6.2
Bio4	Temperature seasonality	—	10	7.2
Bio3	Isothermality	—	8.8	1
Bio10	Mean temperature of warmest quarter	°C	4.3	3.7
Bio15	Precipitation seasonality	—	3.5	2.7
Bio12	Annual precipitation	mm	1.4	2.5
Soil Type	Soil type	—	0.8	0.3

The response curves illustrate how the predicted probability of presence for *N. quadranus* varies along the gradients of the most influential environmental predictors (Figure 3B–F). Bio6 predicted probability of presence approached zero at temperatures above 0°C, increased to a peak around −5°C, and then declined sharply at higher values. Suitability increased sharply with slope, from very low levels on flat terrain (< 1°) to a peak at approximately 3°, and then declined on steeper slopes. Bio2 showed a unimodal relationship with highest suitability around 8°C–8.5°C. Bio4 exhibited a unimodal pattern in the model, with the highest suitability centered at about 800, and decreased toward both lower and higher seasonality values. Bio12 showed higher suitability within the range of 800–1200 mm.

### 3.2 Potential Suitable Habitat for the *N. quadranus*

**FIGURE 5:**
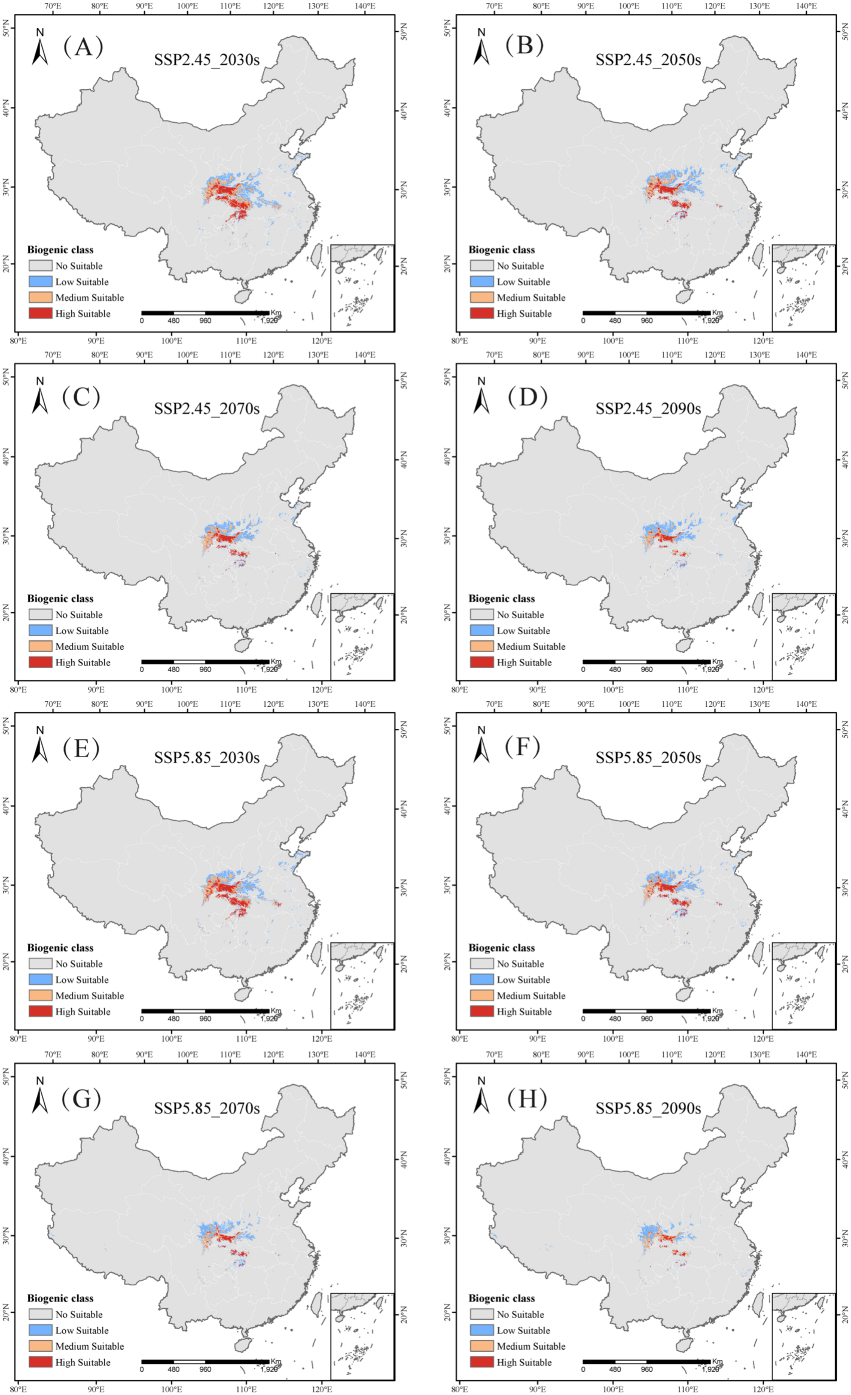
Spatial distribution of the potential suitable habitat of *N. quadranus* under different future climate periods. (A) 2030s (2021–2040) under SSP2‐4.5; (B) 2050s (2041–2060) under SSP2‐4.5; (C) 2070s (2061–2080) under SSP2‐4.5; (D) 2090s (2081–2100) under SSP2‐4.5; (E) 2030s (2021–2040) under SSP5‐8.5; (F) 2050s (2041–2060) under SSP5‐8.5; (G) 2070s (2061–2080) under SSP5‐8.5; (H) 2090s (2081–2100) under SSP5‐8.5.

The current and future potential suitable habitats of *N. quadranus* are illustrated in Figure S1A and Figure 5. A comparison of current habitat suitability between the optimized Maxent and ensemble modeling framework showed a highly similar spatial distribution pattern, with suitable areas concentrated in the Qinling‐Daba Mountains (Figure S1). Under present climatic conditions, the species is mainly distributed across several provinces in central and southwestern China, including Gansu, Chongqing, Shaanxi, Sichuan, Henan, Hubei, Guizhou, Anhui, Shandong, Zhejiang, and Hunan. The quantified suitable habitat areas under different climate scenarios and time periods are summarized in Table S2. At present, the total area of suitable habitat is estimated at 39.95 × 10^4^ km^2^, with low‐, medium‐, and high‐habitat suitability areas accounting for 19.58 × 10^4^ km^2^, 13.15 × 10^4^ km^2^, and 7.22 × 10^4^ km^2^, respectively. The medium‐ and high‐suitability habitats are concentrated in the montane regions of Gansu, Chongqing, Shaanxi, Sichuan, and Henan.

Based on the MTSPS threshold, suitable and nonsuitable areas were delineated from the Maxent modeling outputs. Potential distributions for the 2030s, 2050s, 2070s, and 2090s under the SSP2‐4.5 and SSP5‐8.5 scenarios were subsequently projected and compared with the current distribution patterns (Figure 5; Table S2). Under both scenarios, the total suitable area declines progressively over time, with a more severe and earlier reduction under SSP5‐8.5. Low‐suitability areas increase slightly only in the 2090s under SSP2‐4.5, while medium‐ and high‐suitability regions experience losses throughout the century.

**FIGURE 6:**
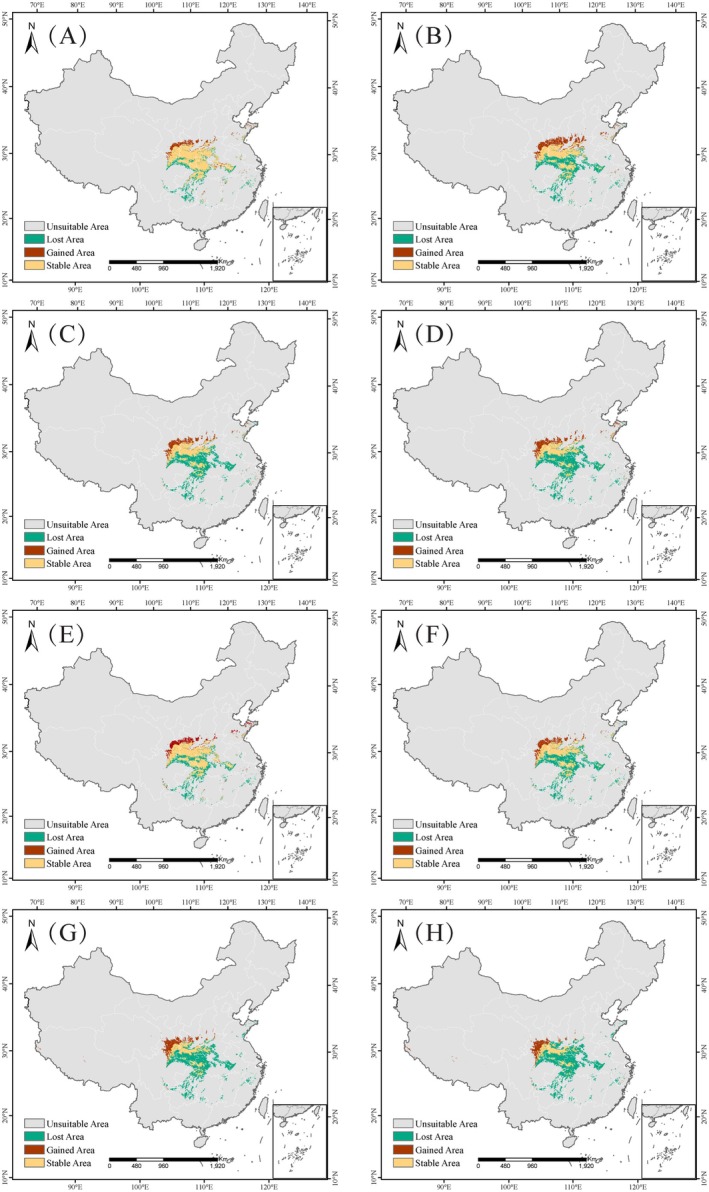
Changes in the spatial pattern of potential suitable habitats of *N. quadranus* under future climate scenarios relative to the current distribution. (A) 2030s (2021–2040) under SSP2‐4.5; (B) 2050s (2041–2060) under SSP2‐4.5; (C) 2070s (2061–2080) under SSP2‐4.5; (D) 2090s (2081–2100) under SSP2‐4.5; (E) 2030s (2021–2040) under SSP5‐8.5; (F) 2050s (2041–2060) under SSP5‐8.5; (G) 2070s (2061–2080) under SSP5‐8.5; (H) 2090s (2081–2100) under SSP5‐8.5.

Based on the current potential distribution, we analyzed the changes in the spatial pattern of future suitable habitats (Figure 6; Table S3). Stable areas decrease under both scenarios, whereas contracted regions (currently suitable but projected to become unsuitable) are consistently larger than expanded regions. This disparity is more pronounced under SSP5‐8.5, where lost areas are larger than newly gained areas (Figure S2).

### 3.3 Geographical Migration of the Distribution Centroid

**FIGURE 7:**
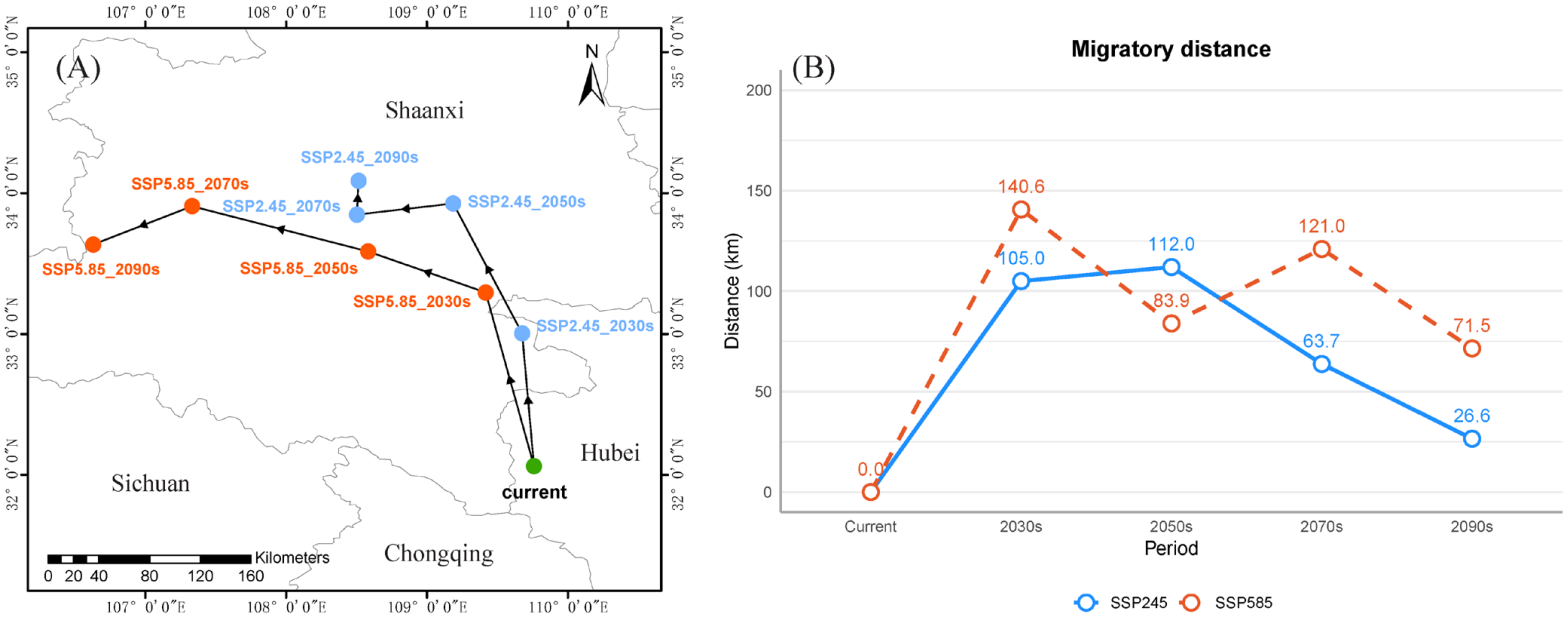
Shift of the distribution centroid of the potential suitable habitat of *N. quadranus* . (A) Shift pathways of habitat centroids; (B) Temporal changes in centroid shift distance.

The geographical centroid of the potential suitable habitat for *N. quadranus* exhibits a clear northwestward migration trend under both future climate scenarios (Figure 7). The centroid coordinates and migration distances across different future periods are summarized in Table S4. Currently, the centroid is located in Hubei Province (32.063° N, 109.759° E).

Under the medium‐emission scenario (SSP2‐4.5), the centroid progressively shifts northwestward. By the 2030s, it moves into Shaanxi Province, representing a northward displacement of 105.0 km. The centroid continues shifting northwestward, reaching a cumulative distance of 217.0 km by the 2050s and 280.7 km by the 2070s. By the 2090s, the cumulative displacement reaches 307.3 km. Under the high‐emission scenario (SSP5‐8.5), the centroid follows a similar directional path but with much larger distances. By the 2030s, it has shifted 140.6 km. It continues moving northwestward, with cumulative distances of 224.8 km by the 2050s, 345.8 km by the 2070s, and 417.3 km by the 2090s.

## 4 Discussion

### 4.1 Model Evaluation and Dominant Predictor Variables

In order to deal with multicollinearity issues, we adopted a stepwise two‐stage variable selection procedure. First, we conducted a correlation filter based on Pearson's coefficients. Second, we further screened out some redundant covariates according to whether they make sense from a biological perspective. Finally, the dimensionality of explanatory variables was reduced from the original 27 to nine ecologically interpretable independent variables. Thus, this selection process ensures that the importance values generated by Maxent are ecologically meaningful rather than spurious. These robust predictive results support our interpretation of the climatic and environmental constraints that potentially limit the distribution of this species (Guisan et al. 2017). Furthermore, we used ENMTools to reduce spatial sampling bias by restricting the background calibration extent and adjusting sample weights. This procedure is a particularly important preprocessing step for cluster‐distributed species. Consequently, these modifications more accurately reflected the accessible environmental space of the species and prevented the model from being skewed toward sampling hotspot areas (Boria et al. 2014; Kramer‐Schadt et al. 2013).

The Maxent model showed excellent predictive performance (AUC = 0.977), surpassing the commonly accepted threshold for high model reliability (Elith et al. 2011; Phillips et al. 2006). Although AUC values are relatively high here, we need to be aware that performance metrics measure statistical separation rather than ecological causality directly (Lobo et al. 2008). To enhance the accuracy of statistical relationships between species and environmental variables, we relied on rigorously screened presence data and ecologically relevant predictors. Furthermore, we systematically tuned the model in ENMeval by adjusting the combinations of RM and FC, rather than relying on default algorithms. The final optimized parameters (RM = 1.0, FC = LQHP) were selected by ENMeval based on the AICc criterion. This combination balances goodness‐of‐fit and model complexity, thereby reducing the risk of overfitting while retaining ecological interpretability (Warren and Seifert 2011). Consequently, the optimized model is expected to yield response curves that are less overfitted and may offer improved transferability, thereby providing useful insights into relationships of species and environment.

Using biomod2 as an additional tool for spatial consistency validation also provided important cross‐validation for the optimized Maxent results. The high spatial coincidence between our optimized Maxent model and the ensemble consensus from biomod2 further reinforces the robustness of the predictions. Such spatial coincidence supports the robustness of the predicted suitable habitat patterns. Given the high agreement on the importance of Bio6 across all five algorithms, we conclude that low winter temperature is strongly associated with the distribution of species (Elith and Graham 2009).

### 4.2 Climatic and Environmental Predictor of Distribution

Temperature‐related variables were identified as the most important predictors of *N. quadranus* distribution, consistent with previous studies on the thermal sensitivity of amphibians (Duarte et al. 2012; Li et al. 2013). Among all environmental predictors identified in this study, Bio6 was identified as the most important predictor, suggesting a statistical association between winter minimum temperature and the potential distribution of this species. The response curve for Bio6 revealed a unimodal pattern, with the highest predicted habitat suitability occurring at approximately −5°C when all other variables were held at their mean values. Bio4, Bio3, and Bio2 were also important predictors, associated with the thermal range and environmental stability related to the distribution of this species. Optimal suitability is observed at response curve peaks of approximately 800 for Bio4, 27 for Bio3, and 8.3°C for Bio2. These specific thermal thresholds reflect pronounced seasonal variations and moderate diurnal fluctuations, characteristic of a temperate mountain climate (Körner 2007). The response patterns observed for these temperature‐related variables suggest that the potential distribution of this species is likely constrained by thermal regimes characteristic of temperate montane environments. The strong statistical association between temperature‐related variables and the potential distribution of the species is considered ecologically plausible (Wei et al. 2025). This is because the physiological processes of ectothermic species are known to be controlled by environmental temperature fluctuations, including metabolic regulation and overwintering (Park and Do 2025). Prolonged exposure to temperatures below physiological tolerances may expose ectothermic species to risks of freezing injury, energy exhaustion, and mortality (Storey and Storey 2017). Conversely, abnormally warm winters may prevent the reduction of metabolic rates. Due to the scarcity of food resources during this period, their fat reserves will be rapidly depleted. As a result, when they wake up in spring, their bodies will be extremely weak, and some may even die before the end of winter (Benard 2015; Reading 2007). For amphibians living in temperate regions, prolonged exposure to low temperatures is a necessary prerequisite for the maturation of their gonads, which is a process similar to the “vernalization” in plants. Without this crucial low‐temperature stimulus, the hormone pathways necessary for reproduction cannot be activated, ultimately leading to reproductive failure (Santana et al. 2015). Under future climate warming scenarios, rising winter temperatures will likely trigger elevated metabolic costs or disrupted reproductive phenology, ultimately threatening the long‐term persistence of these populations.

Slope, explaining more than 10% of the model prediction, had the highest contribution among the nonclimatic predictors. The high PC and PI of slope suggest a strong association with habitat suitability for *N. quadranus* . This association may partly reflect the role of topography in mediating local climatic conditions, consistent with previous evidence that topography moderates climatic effects in montane landscapes (Scheffers et al. 2014).

### 4.3 Spatiotemporal Changes Under Climate Change

Future climate projections indicate a pronounced decline in suitable habitat for *N. quadranus* under two emission scenarios, with the extent of stable habitat areas progressively diminishing over time. Gained areas cannot compensate for the loss of stable habitats, resulting in a continuous decline in the species' potential geographic distribution. Under the high‐emission scenario (SSP5‐8.5), the stable habitat area is projected to shrink to 9.22 × 10^4^ km^2^ by the 2090s, a reduction of approximately 63% compared to the 2030s under the same scenario. Meta‐analyses indicate that when temperature change adds to a long‐term warming trend, extinction risk can increase by up to 40% (Mathes et al. 2025). Therefore, the substantial reduction in stable habitat for *N. quadranus* under the SSP5‐8.5 scenario may increase the probability of population decline and potential regional extirpation.

From the perspective of centroid movement distance, the centroid of suitable habitat for *N. quadranus* is projected to shift northwestward by 307.3 km under the SSP2‐4.5 scenario and 417.3 km under the SSP5‐8.5 scenario. Aquatic‐breeding amphibians are often characterized as having limited overland dispersal ability. Their movement is constrained by topographic conditions, making it difficult for them to cross mountainous areas with steep slopes and large elevation changes. Tracking data from a congeneric species, *Nanorana vicina* , indicate that its daily movement is typically confined to a range of just a few meters within stream channels, with almost no overland migration (Nawani et al. 2024). The shift distance of suitable habitat far exceeds the natural dispersal range of this species. Furthermore, *N. quadranus* may encounter anthropogenic barriers—such as roads, agricultural lands, and urban areas—along potential migration pathways, which may further exacerbate the difficulty of shifting toward suitable habitats.

From the perspective of centroid movement direction, the suitable habitat of *N. quadranus* is projected to shift northwestward in the future. This aligns with observations in *Quasipaa spinosa* (Hou et al. 2023). Under future climate warming scenarios, winter warming effects are more pronounced at high latitudes, and areas previously unsuitable due to low winter temperatures may become potentially suitable (Pakanen et al. 2018). However, a northwesterly shift in predicted climatically suitable areas does not necessarily imply that species can actually achieve such range shifts. Climate warming may also trigger multiple secondary stressors, such as increased extreme summer heat and altered precipitation patterns, which may reduce the quality of existing suitable habitats and ultimately lead to a net loss of suitable areas. The overall contraction of suitable habitat predicted by the model is consistent with the potential impacts of these multiple stressors. In addition, although the predicted centroid endpoint remains within a semihumid region, further northwestward expansion of suitable habitat would gradually enter the semiarid region of the Northwest China. This area lacks the hydrological conditions necessary for the survival of *N. quadranus* , thereby further hindering its movement toward suitable habitats.

### 4.4 Conservation Implications

Based on our model projections, the suitable habitat of *N. quadranus* is expected to contract significantly under future climate stress. Therefore, this study proposes the following conservation measures at a macro level to provide a reference for the protection of this species.

Prioritizing suitable habitats and refining management measures. Regions identified by the models as persistently suitable across all future scenarios should be regarded as priority conservation areas and integrated into the existing protected area planning system (Hannah et al. 2007). Large‐scale climate models often overlook the buffering effects of complex terrain on climate. River valleys in the Qinling‐Daba Mountains may form microhabitat, providing cooler and wetter microhabitats for species than the surrounding environment (Morelli et al. 2016). Therefore, future conservation measures should not rely solely on macro‐model results but must be combined with field surveys to verify and identify these microhabitats with high topographic complexity. Meanwhile, disturbances such as hydropower development and tourism activities should be reduced (Zhou et al. 2026; Tan et al. 2025). Long‐term monitoring and iterative model correction are also important (Wang et al. 2024). Given the dynamic and nonlinear trajectory of future climate change, static conservation measures are insufficient to address emerging uncertainties. We therefore advocate for an adaptive management framework structured around a continuous monitoring–feedback–adjustment cycle (Souza Oliveira et al. 2025). Specifically, we propose establishing long‐term monitoring plots across the species' core distribution range. Monitoring should extend beyond simple presence‐absence data to include key life‐history traits such as reproductive rate and metamorphosis rate. This would enable early detection of population declines.

The predicted centroid shift distance in this study is much greater than the natural dispersal distance of *N. quadranus* . Therefore, assisted migration could be considered a potential option if future monitoring data indicate a persistent population decline, but it would require careful evaluation (Hoegh‐Guldberg et al. 2008). However, moving a species to areas outside its historical range is a highly complex conservation intervention, involving substantial ecological, genetic, and management risks, including unintended hybridization, disease introduction, and in severe cases, disruption of local ecosystems. Such interventions should therefore only be considered under extreme circumstances and must be preceded by rigorous risk assessment and long‐term monitoring. We emphasize that habitat protection and restoration, along with enhancing the connectivity of suitable habitats, remain the primary conservation measures.

## 5 Conclusions

In this study, an optimized maximum entropy model (Maxent) combined with Geographic Information System (GIS) technology was used to predict the present and future suitable habitat for *N. quadranus* under the SSP2‐4.5 and SSP5‐8.5 scenarios. Concurrently, the biomod2 multialgorithm ensemble framework was employed for independent spatial validation of the optimized Maxent predictions. Temperature‐related variables, especially Bio6 (Minimum Temperature of Coldest Month), were identified as the most important predictors of its distribution, followed by Bio4 (Temperature Seasonality), Bio3 (Isothermality), and Slope. Compared with water limitation, temperature and topography appear to be the primary factors strongly associated with the distribution range of *N. quadranus* . Future climate projections show that suitable habitat will be significantly reduced and shift northwestward. According to SSP2‐4.5 and SSP5‐8.5 scenarios, the centroid is projected to shift by 307.3 km and 417.3 km respectively, by the end of the century. Under future climate warming scenarios, the conservation of *N. quadranus* should prioritize securing persistently suitable habitats and maintaining the natural connectivity of these environments. This study provides a scientific basis for the conservation planning of this endemic species and offers a reference for the protection of other amphibians inhabiting cold‐water stream habitats in the Qinling‐Daba Mountains.

## Author Contributions

**Yan Huang:** conceptualization (equal), funding acquisition (equal), project administration (equal), supervision (equal), writing – review and editing (equal). **Yuan Sun:** conceptualization (equal), formal analysis (equal), methodology (equal), software (equal), visualization (equal), writing – original draft (equal). **Lulu Lyu:** data curation (equal), investigation (equal). **Jie Peng:** investigation (equal), resources (equal).

## Funding

This research was funded by the National Natural Science Foundation of China (32270461) and Natural Science Research and Innovation Team Project of China West Normal University (KCXTD2024‐6).

## Ethics Statement

The study was conducted according to the guidelines of the Declaration of Sichuan and approved by the Ethics Committee of China West Normal University. All methods for capturing and handling animals used in this study were approved by the Institutional Animal Care and Use Committee (IACUC) at China West Normal University (CWNU2019D012).

## Consent

The authors have nothing to report.

## Conflicts of Interest

The authors declare no conflicts of interest.

## Supporting information


**Code S1.** biomod2.

**Figure S1:** Comparison of current habitat suitability for *N. quadranus* between the optimized Maxent and ensemble modeling frameworks. (A) Predicted distribution based on the optimized Maxent model; (B) Predicted distribution based on the ensemble consensus (EMwmean) from the biomod2 framework. The discrete suitability classes (High, Medium, Low, and No Suitability) were delineated using the MTSPS threshold.

**Figure S2:** Spatiotemporal dynamics of suitable habitat under future climate scenarios. (A) Composition and trends of suitable habitat change under SSP2‐4.5; (B) Composition and trends of suitable habitat change under SSP5‐8.5.pdf.

**Table S1:** All Candidate Models Evaluation Results.csv.

**Table S2:** Changes in the area of potential suitable habitats of *N. quadranus* under current and future climate scenarios (SSP2‐4.5 and SSP5‐8.5) across different time periods. Habitat suitability was classified into low, medium, and high categories based on the MTSPS threshold. Values are expressed as area (×10^4^ km^2^), and percentage change represents variation relative to the preceding period.csv.

**Table S3:** Changes in the area of potential suitable habitats of *N. quadranus* under current and future climate scenarios (SSP2‐4.5 and SSP5‐8.5) across different time periods based on the MTSPS threshold. Values are expressed as area (km^2^), and percentage change represents variation relative to the preceding period.csv.

**Table S4:** Centroid coordinates and range shift magnitude.csv.

**Table S5:** ece374131‐sup‐0008‐TableS5.csv. *nanorana_quadranus*_occurrence_data.csv.

## Data Availability

The data supporting the results are available in a public repository at: https://doi.org/10.5061/dryad.34tmpg4zw.
